# A Gas Pressure Prediction Model of the Excavation Face Based on Gas Emission

**DOI:** 10.3390/ijerph19084891

**Published:** 2022-04-18

**Authors:** Liang Chen, Qi Liu

**Affiliations:** School of Energy & Environment Engineering, Zhongyuan University of Technology, Zhengzhou 450007, China

**Keywords:** gas pressure, mining, coal and gas outburst, warning

## Abstract

Gas pressure is one of the important factors related to the occurrence of coal and gas outburst disasters. The accurate gas pressure forecasting is of significance for the prevention and control of a gas disaster. In this work, a gas pressure prediction model based on the sources of gas emissions was established. The verified results show that the predicted gas pressure was roughly consistent with the actual situation. This model could meet the needs of engineering projects. Coal and gas outburst dynamic phenomenon are successfully predicted in an engineering application using the model. Overall, the prediction of coal and gas outburst using the gas pressure model achieves a continuous and dynamic effect. The model can overcome both the static and sampling shortcomings of traditional methods and solve the difficulty of coal and gas outburst prediction at the excavation face. With its broad applicability and potential prospects, the model is of great importance for guiding gas drainage, and the prevention of coal and gas outburst disasters.

## 1. Introduction

Coal and gas outbursts occurring in the coal mining process are dynamic phenomena accompanied by great hazards [1]. The risk of this type of disaster exists in almost all of the main coal-producing countries in the world. Among them, approximately one-third have occurred in China and it is considered the primary hazard with respect to coalmine safety [2]. With mining depth and intensity continuously increasing and geological conditions gradually becoming more complicated, coal and gas outburst disasters are causing serious problems all over the world [3,4] Gas pressure is one of the important parameters for studying gas emissions and predicting coal and gas outburst risks [5,6]. Therefore, the accurate prediction of gas pressure is an urgent priority.

Current prediction techniques are unreliable and unexpected outburst incidents that can result in fatalities are a major concern for underground coal mining [7,8]. Conventional methods for the prediction of coal and gas outbursts are mainly based on coal seam drilling in advance of mining. These methods predict a coal and gas outburst by measuring the initial velocity of a gas emission from boreholes, the measure of the drill cuttings weight and other parameters. Their implementation has reduced the occurrence of coal and gas outbursts to a certain extent, but an outburst can still occur when the indexes are below the warning criteria. This is primarily because coal and gas outburst are very complex [9,10].

Some unconventional prediction methods, such as the method of methane concentration and *V*_30_ [1], the method of coal desorption property *V*_1_ [11], gas content [12], gas emission [13] and gas dilatation energy [14], were proposed and applied in field trials to predict a coal and gas outburst disaster and some results were obtained. Geophysical methods such as those using electromagnetic radiation, micro seismic and acoustic emissions [15,16,17] have also been successfully applied in some coal mines.

Some researchers designed a gas-measurement-tube to measure gas pressure and its changes in outburst-prone regions and they calculated the permeability of coalbeds nearby to the working face, thus, they were able to further evaluate the risk of coal and gas outburst [18]. Moreover, underground horizontal boreholes were utilized to test the permeability and stress, and the strength and adsorption properties of the sampled coal were measured in their laboratory based on the adopted Monte Carlo technique to assess the risk of outbursts [19].

Although there have been many conventional and unconventional prediction methods, gas outburst has not been completely eliminated. Gas pressure is one of the important indicators reflecting a coal and gas outburst hazard. Therefore, compared with the conventional and unconventional methods, predicting coal and gas outbursts using gas pressure has a higher accuracy and universality. The traditional technique based on gas pressure used in outburst prediction utilizes drilling, sealing and measurement, but the need for such a laborious process has led some researchers to investigate alternative methods of prediction.

Orthogonal tests were designed and conducted to investigate the influences of initial gas pressure, coal particle size and outburst mouth diameter on a coal and gas outburst. The critical initial gas pressure and gas content were calculated as a reference to predict the coal and gas outburst danger [20]. A method for determining the law governing stress and gas pressure coupling was proposed based on stress–strain curves obtained through triaxial loading tests. When the stress exceeds the compressive strength of the coal, gas pressure begins to decrease with that stress. Thus, an increasing stress and gas pressure are negatively correlated [21]. A new apparatus (LSTT) was developed to conduct a simulated experiment. In the process of a coal and gas outburst, the gas enthalpy of the gas and the elastic potential of the coal are the main energy sources [22].

Coal seam dynamic disasters caused by gas (CO_2_) injection displacement were studied. The results of the gas injection displacement experiment revealed that CO_2_ plays a significant role in replacing CH_4_, but a large amount of CO_2_ is adsorbed into the coal seam before the CH_4_ can be completely desorbed, which in turn raises the outburst risk of the coal seam [23]. Considering the effect of coal damage and the environmental pressure change for desorption, a method to calculate the gas expansion energy for an outburst initiation is provided [24].

The safety line method for predicting gas pressure also meets the needs of some coal and gas outburst-susceptible mines [25]. Moreover, the development of numerical simulation technology provides a new tool for predicting the gas pressure. The finite difference method was applied to study the distribution of gas pressure and the characteristics of gas emission from the areas around the excavation face and roadway [26]), while the distribution of gas pressure ahead of the tunneling was analyzed when the gas content was assumed to be constant [27]. Although these gas pressure prediction methods meet some field requirements, the dynamic behavior of underground coalbed gas pressure cannot be completely reflected, therefore they are inadequate for the accurate prediction of coal and gas outburst.

A continuous dynamic model of gas pressure is necessary to provide early-warning of a coal and gas outburst. The gas emission during a roadway excavation is the result of interactions between the geological conditions and coalbed occurrence, consistent with gas pressure impacting factors. Gas monitoring systems have wide application in coal mines and offer online support, therefore, they can be beneficial to predicting gas pressure using gas emission. It is believed that such a method could solve the gas pressure prediction difficulty that has troubled coalmines for many years. Based on this, the research presents a continuous dynamic model to predict gas pressure at the excavation face, verified and applied in a practical engineering case.

## 2. Gas Pressure Model Based on Gas Emission

Coal seam gas emission is driven by the gas pressure gradient through the coal pore fracture to the workspace. It is related to desorption, adsorption, diffusion, convective flow, etc., and these are closely related to gas pressure. Thus, a gas pressure prediction model according to the sources of the working face gas emission can be established by considering the fluid–solid coupling process.

### 2.1. Gas Emission Model

There are two sources of gas emission from the excavation face. One comes from collapsed coal and the other comes from the coal walls [28,29]. The intensity of gas emission at the excavation face can be described by:

*Q* = *Q_c_* + *Q_w_* (1)

where *Q_c_* and *Q_w_* are the gas emission intensities from the collapsed coal and the coal walls, respectively, m^3^/min.

Gas from the coal walls is continuously supplied by the coalbed and affected by the fractures formed by underground pressure and coal damage, as well as mining procedures. Therefore, the intensity of the gas emission and its attenuation fluctuates greatly with changes in the underground pressure and crack production, but in general, it obeys the law of exponential decay [30]. By contrast, gas emission from collapsed coal is not affected by its supply source and underground pressure and it does not fluctuate in its decay process.

#### 2.1.1. Intensity of Gas Emission from Collapsed Coal

The intensity of the gas emission from collapsed coal per ton per minute is:

(2) $$Q_{1}=Q_{0}e^{-\beta_{1}t_{1}}$$

where *Q*_1_ and *Q*_0_ are the intensities of the gas emission from collapsed coal at time *t*_1_ and at the initial time, respectively, m^3^/(t.min); $\beta_{1}$ is the decay coefficient of the collapsed coal gas, min^−1^; and *t*_1_ is the time that the collapsed coal remains at the face, min.

The total intensity of gas emission from the collapsed coal *Q_c_* becomes:

(3) $$Q_{c}=G_{c}Q_{0}e^{-\beta_{1}t_{1}}=XS_{cs}\gamma Q_{0}e^{-\beta_{1}t_{1}}$$

where *G_c_* is the amount of collapsed coal from the mining, t; *X* is the exposed area at the face, m; *S_cs_* is the cross section of the roadway, m^2^; and γ is the bulk density of the coal, t/m^3^.

#### 2.1.2. Intensity of Gas Emission from the Coal Wall

(4) $$Q_{f}=qS_{cs}e^{-\beta_{2}t_{2}}$$

where $\beta_{2}$ is the decay coefficient of the coal wall gas, min^−1^; and *t*_2_ is the exposure time of the coal wall, min.

Likewise, the intensity of the gas emission from a unit area of the roadway wall is:

(5) $$Q_{3}=qe^{-\beta_{2}t_{2}}$$

where $Q_{3}$ is the amount of gas emission from a unit area of roadway wall at time *t*_2_, m^3^/ (m^2^.min).

Supposing that a small length segment along the roadway is *dl* and the rate of gas emission around the roadway wall along *dl* obeys Equation (5), the amount of gas emission from *dL* at *t*_2_ is:

(6) $$dQ_{3}=qe^{-\beta_{2}t_{2}}Adl$$

where *A* is the perimeter of the coal wall, m.

After excavating *X* m, at location *L* far from the roadway head, the amount of gas emission from the roadway wall *Q_r_* is the integral of Equation (6) from the roadway head to location *L*, that is:

(7) $$Q_{r}=\int_{0}^{L+X}qe^{-\beta_{2}t_{2}}Adl=qA(L+X)e^{-\beta_{2}t_{2}}$$

According to Equations (4) and (7), the intensity of gas emission from the coal wall is:

(8) $$Q_{w}=q[S_{cs}e^{-\beta_{2}t_{2}}+A(L+X)e^{-\beta_{2}t_{2}}]$$

From Equation (8), it can be seen that gas emission from the coal wall is closely related to the intensity of the gas emission per unit area of the coal wall.

To obtain the intensity of the gas emission per unit area of the coal wall, it is assumed that the process of the coalbed gas migration is an isothermal process, where the free gas is an ideal gas complying with the ideal gas equation of state; and that the coal is a continuous medium, the plastic deformation of the gas bearing coal is small, and the gas flow in the coal wall is unidirectional and steady.

The gas adsorption obeys the Langmuir equation [28,31] and the content of gas can be expressed as:

(9) $$X_{m}=\frac{abp}{1+bp}+Bnp$$

where *X_m_* is the content of the gas per unit coal mass, m^3^/t; *a* is the limiting adsorption amount of coal, m^3^/t; *b* is the adsorption equilibrium constant, MPa^−1^; *p* is the coalbed gas pressure, MPa; *n* is the porosity of the coal; *B = T*_0_/(*Tp_0_ξρ*), *T*_0_ is the absolute temperature (under standard conditions, *T*_0_ = 273 *K)*; *T* is the gas temperature, K; *p*_0_ is the atmospheric pressure (under standard conditions, *p*_0_ = 0.101325 MPa); ξ is the gas compression factor; and *ρ* is the apparent density of the coal, t/m^3^.

The gas flow in the coal is determined by Darcy’s law [28,31]

(10) $$u=-\frac{k}{\mu }\frac{\partial p}{\partial x}$$

where *u* is the velocity of the gas flow, m/s; *k* is the permeability of the coalbed, m^2^; *μ* is the coefficient of dynamic viscosity of the gas, MPa·s; and ∂*p*/∂*x* is the gradient of gas pressure, MPa/m. According to the ideal gas law, the velocity of the gas flow was converted to a volume flux. Hence, Equation (10) can be written as:

(11) $$Q_{v}=-\lambda \frac{\partial p^{2}}{\partial x}$$

where *Q_v_* is the gas volume flux per day, m^3^/ (m^2^.d); and λ is the coal permeability ratio, $\lambda =Ck/2\mu p_{0}$, m^2^/ (MPa^2^.d), where *C* is the unit conversion factor of seconds to days.

After introducing the volume flux per minute *q*, $Q_{v}$ can be written as $Q_{v}=q/1440$.

Hence, Equation (11) can be written as:

(12) $$\frac{q}{1440}=-\frac{Ck}{2\mu p_{0}}\frac{\partial p^{2}}{\partial x}$$

Therefore, putting Equation (12) into Equation (8), one finds the intensity of the gas emission from the coal wall to be:

(13) $$Q_{w}=[S_{cs}e^{-\beta_{2}t_{2}}+A(L+X)e^{-\beta_{2}t_{2}}](-\frac{720Ck}{\mu p_{0}}\frac{\partial p^{2}}{\partial x})$$

#### 2.1.3. Model for Continuous Gas Emission

By combining Equations (1), (3) and (13) one can obtain the model of continuous gas emission from the excavation face as follows:

(14) $$Q=XS_{cs}\gamma Q_{0}e^{-\beta_{1}t_{1}}+[S_{cs}e^{-\beta_{2}t_{2}}+A(L+X)e^{-\beta_{2}t_{2}}](-\frac{720Ck}{\mu p_{0}}\frac{\partial p^{2}}{\partial x})$$

### 2.2. Model for Gas Pressure Prediction

According to the inversion of the model of continuous gas emission, the model for gas pressure prediction can be expressed as:

(15) $$p=\sqrt{\frac{\mu p_{0}(Q-XS_{cs}\gamma Q_{0}e^{-\beta_{1}t_{1}})l}{720Ck[S_{cs}e^{-\beta_{2}t_{2}}+A(L+X)e^{-\beta_{2}t_{2}}]}}$$

where *l* is the affecting scope of gas seepage, m.

## 3. Verification of Gas Pressure Model

**Figure 1:**
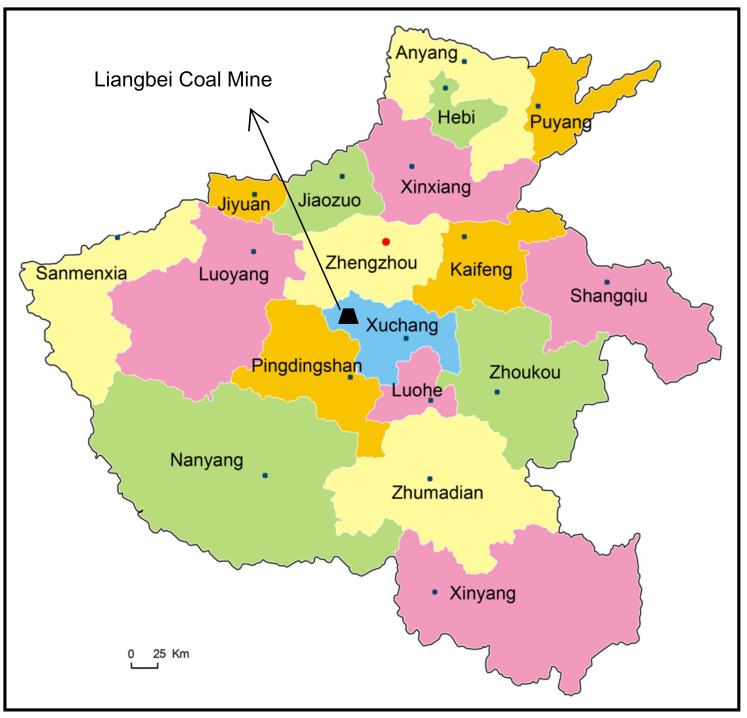
Geographical location of Liangbei Coal Mine.

The gas pressure prediction model was verified in the Liangbei Coal Mine. Liangbei Coal Mine is located 37 km west of Xuchang City, Henan Province, China, as shown in Figure 1. It belongs to the Shenhuo Coal Industry Group. Its annual raw coal output is 900,000 tons. In its production process, the coal mine has had many coal and gas outbursts, extrusions, rib spalling, floor heave and serious deformations of the roof and both sides of the roadway.

### 3.1. Geological Background

**Figure 2:**
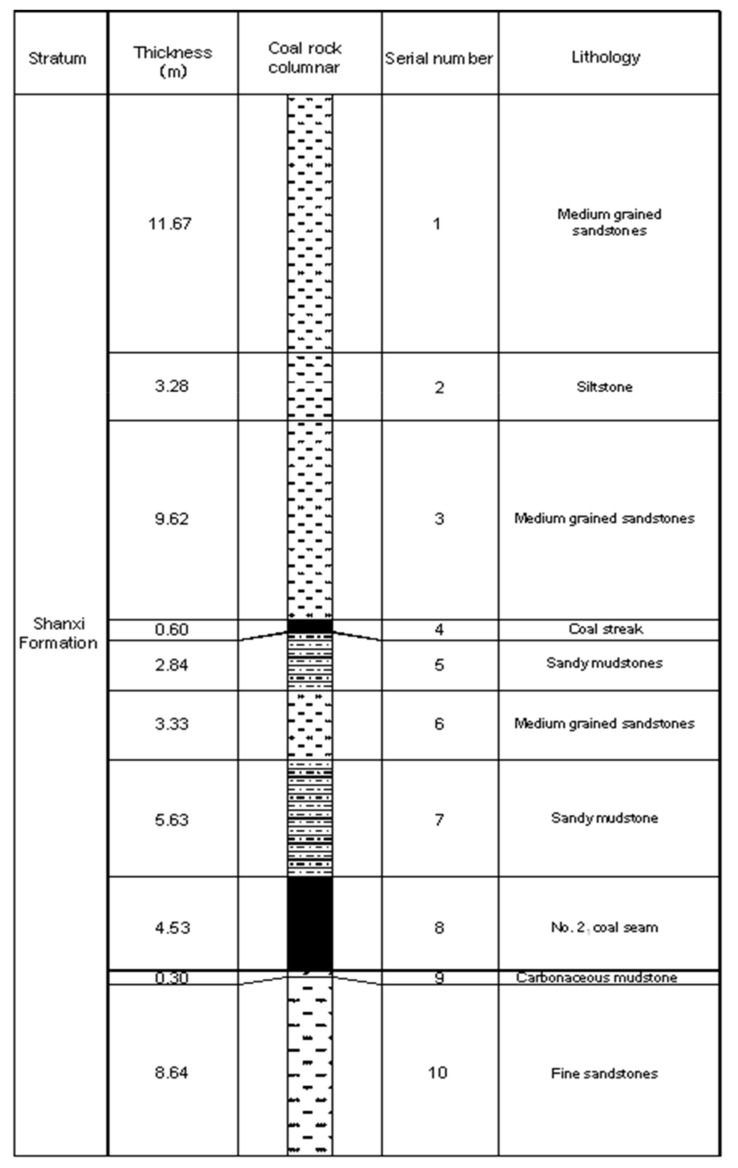
Comprehensive stratigraphic column of the Shanxi Formation strata.

Currently, the main coalbed of the Liangbei Coal Mine is the No. 2_1_ coalbed located at the bottom of the Permian Shanxi Formation. Figure 2 shows the comprehensive stratigraphic column of the Shanxi Formation of the No. 11131 excavating face. The No. 2_1_ coalbed has a stable occurrence and a relatively simple geological structure. Its average thickness is 4.53 m, and its average dip is 13°, in the range of 8~15°. Its roof is a 5.63 m thick dark gray sandy mudstone. The mudstone has well-developed horizontal bedding, containing small visible muscovite flakes and rich plant fossil debris. Its immediate floor is 8.64 m thick, dark gray, thin-layered, fine sandstones mixed with muddy strips with wavy bedding, and it contains a large amount of plant fossil fragments. Its original gas pressure was 0.6~3.65 MPa and its gas content about 5.73~13.97 m^3^/t. The attenuation coefficient of the gas flow from the borehole per 100 m into the coalbed is 0.0313~0.2588 d^−1^ and the coal permeability ratio is 0.0011~0.0454 m^2^/MPa^2^·d. According to the Chinese code for coal mine gas drainage, the coalbed is more difficult for gas drainage [32]. The quality of the coal is soft with a Protodyakonov coefficient of 0.15~0.25. Table 1 shows the model’s initial physical parameters.

### 3.2. Verification Result

**Figure 3:**
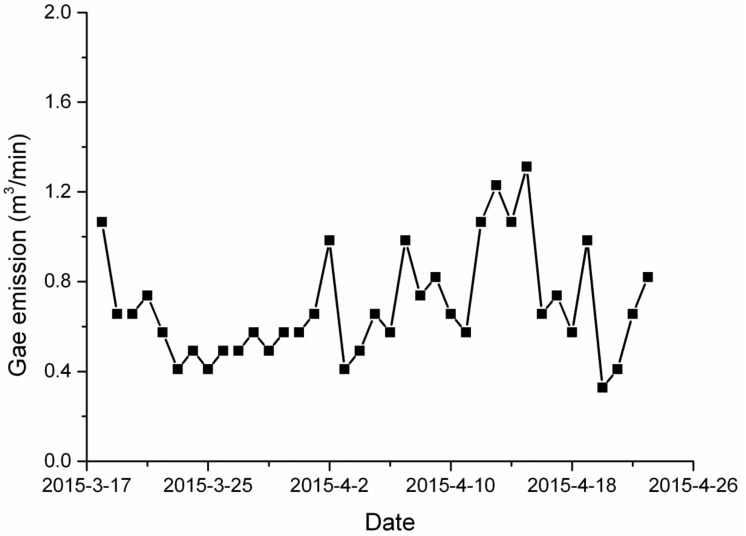
Gas emission from No. 11131 mining face.

Figure 3 shows the intensity of the gas emission from the No. 11131 excavating face of the Liangbei Coal Mine from 18 March to 21 April 2015. From Figure 3, it is clear that the minimal and maximal gas emission rate were 0.33 m^3^/min and 1.31 m^3^/min, respectively.

**Figure 4:**
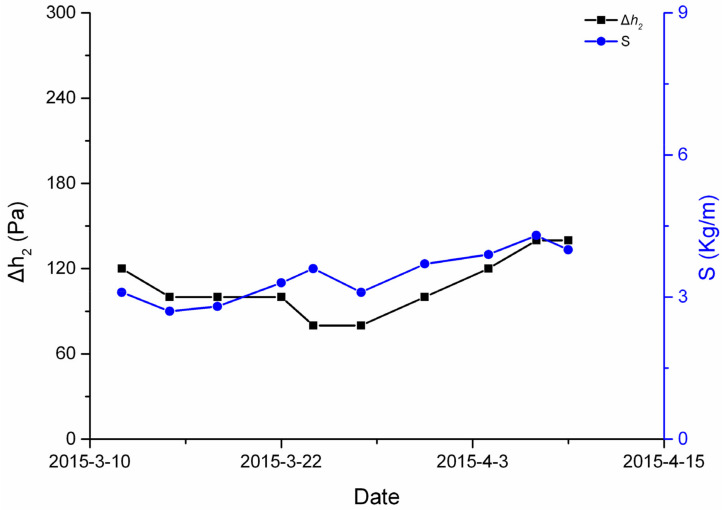
Changes of conventional indicators.

Figure 4 shows the changes in the conventional indicators. From the graphs it is obvious that during this time, the minimal and maximal drill cuttings desorption indexes, Δ*h*_2,_ were 80 Pa and 140 Pa, respectively, and the minimal and maximal drill cuttings weight, *S*, were 2.7 kg/m and 6 kg/m, respectively. One of the conventional indicators was more than the warning criteria of risk. This indicates that there was coal and gas outburst risk and the factors impacting gas emission including the stress, gas pressure as well as the coal’s physical and mechanical properties had changed. This is the main reason for the rapid fluctuation of gas emission during this period.

Measuring gas pressure takes several days or even months. Hence, the borehole gas content was measured on-site and Equation (9) was used to deduce the gas pressure by the gas content. The gas pressure based on the prediction model was verified according to the coal seam gas content.

**Figure 5:**
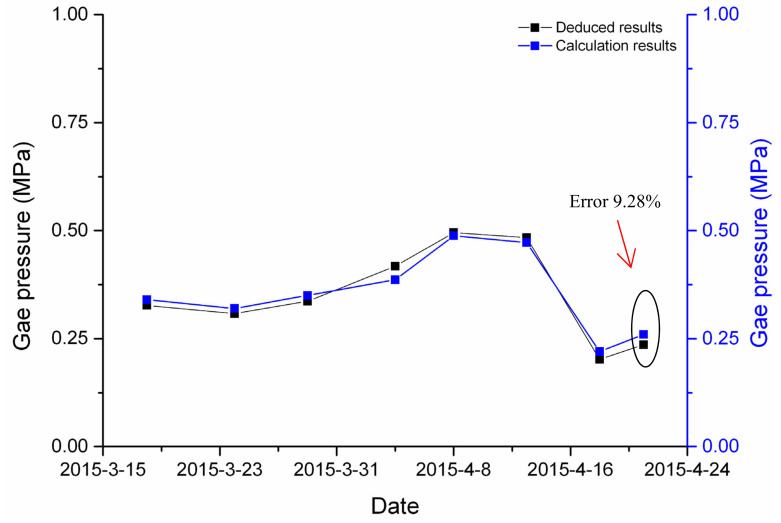
Comparison between calculation and deduced gas pressure results.

Figure 5 shows a comparison between the calculation and deduced gas pressure. It can be seen from the figure that the maximal deviation was 9.28%, indicating that the calculation results were roughly consistent with the deduced results.

The verification of the gas-emission-based gas pressure prediction model clearly indicates that the model has a higher accuracy, making it suitable for engineering needs.

## 4. Engineering Application

Coal and gas outburst events have occurred in the Liangbei Coal Mine several times. For example, on 29 June 1999, a coal and gas outburst occurred in the excavation process of the main crosscut, discharging 180 tons of coal and 18,000 m^3^ of gas; on 8 July 2009, a coal and gas outburst happened during the opening of the No.2_1_ coal seam at the return airway crosscut, ejecting 600 tons of coal and approximately 50,000 m^3^ of gas. During the current production, coal and gas outburst phenomena, such as gas spurting from boreholes and drill-bit suction, have occurred many times. Gas is an important factor causing a coal and gas outburst disaster. In China, gas pressure less than 0.74 MPa or gas content less than 8 m^3^/t is regarded as posing no outburst risk; however, the No.2_1_ coalbed of the Liangbei Coal Mine has a strong outburst risk and low index coal and gas outbursts have occurred several times. A coal and gas outburst is very difficult to predict accurately. In the Henan Province where the Liangbei Coal Mine is located, stricter regulations are necessary. Gas pressure less than 0.6 MPa or gas content less than 6 m^3^/t is regarded as posing no outburst risk. Thus, the application of the new gas pressure prediction method may help solve the coal and gas outburst prediction problem of the Liangbei Coal Mine.

**Figure 6:**
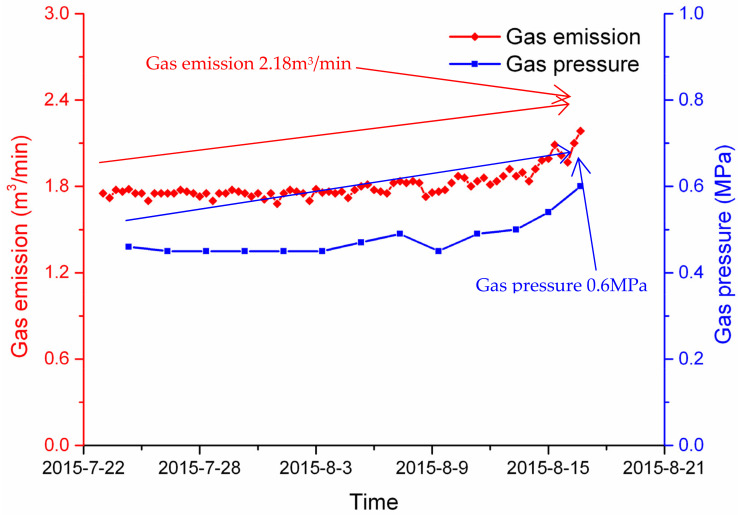
Gas pressure using the prediction model before the dynamic phenomenon occurred.

On 16 August 2015, workers on the 16:00 shift of the Liangbei Coal Mine found that gas emission from the No.11131 excavation face rose slowly from 1.73 to 2.1 m^3^/min from 23 July to 16 August, reaching 2.18 m^3^/min on 16 August, as shown in Figure 6. The predicted gas pressure based on the new model reached 0.6 MPa. Thus, the excavation was stopped for drilling to test the risk and relieve the stress and gas pressure. During the drilling process, a slight borehole-spurting phenomenon occurred; however, impacts on the geological structure and other related factors were not found.

**Figure 7:**
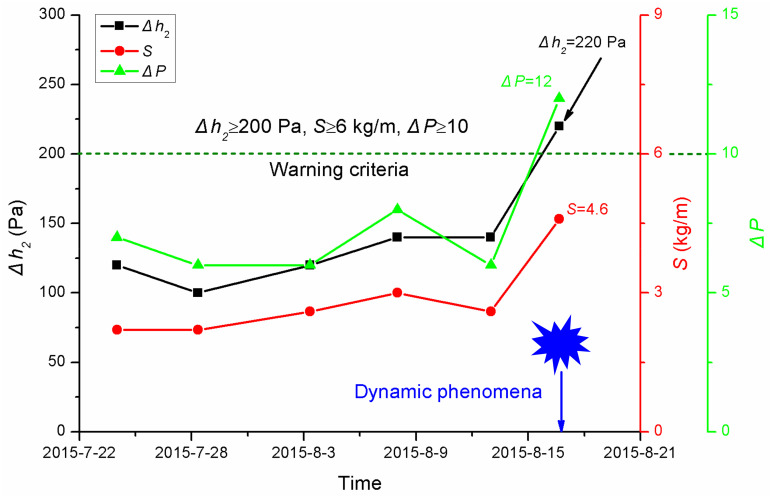
Conventional indicators before the dynamic phenomenon occurred.

The index of gas desorption from drill cuttings, Δ*h*_2_, and the initial velocity of gas emission, Δ*P*, but not the drill cuttings weight, were beyond their warning criteria. Figure 7 shows the conventional indicators measured on 16 August 2015. From Figure 7, it is clear that before 16:00 on 16 August 2015, the index of gas desorption from the drill cuttings, Δ*h*_2_, was 100~140 Pa, the initial velocity of gas emission, Δ*P*, was 6~8 and the cuttings magnitude, *S*, was 2.2~3 kg/m, all of which were less than their critical values for outburst risks.

The above prediction verification proves that the gas-pressure prediction model for coal and gas outburst can be used for continuous and dynamic prediction and it overcomes the static and sampling shortcomings of traditional methods. The new method for coal and gas outburst prediction at the excavation face has advantages over the conventional methods in enabling continuous and dynamic prediction.

## 5. Conclusions

A continuous dynamic prediction model of gas pressure was established in this research. It is based on gas emission. The results according to the prediction model were roughly consistent with the actual situation, with errors in the coalbed gas pressures in the range of 9.28%. The gas pressure prediction model fully considers the factors and has a higher accuracy. Thus, it can meet the needs of engineering.

The gas pressure prediction model was successfully used to predict the coal and gas outburst dynamic phenomenon occurring at the roadway excavation face of the Liangbei Coal Mine. Before the outburst, all of the conventional indicators of the face were below their critical values for outburst risks. This shows that the gas pressure prediction model, as a new method for coal and gas outburst prediction, achieves continuous and dynamic prediction for coal and gas outburst. The model overcomes the static and sampling shortcomings of traditional prediction methods and it is believed that the model has broad applicability and great potential.

## Figures and Tables

**Table 1:** Basic parameters of the model.

Parameter	Value
Elastic modulus of roof and floor rocks	30 GPa
Poisson’s ratio of roof and floor rocks	0.22
Density of roof and floor rocks	2.5 t/m^3^
Internal cohesive force of roof and floor rocks	40 MPa
Internal friction angle of roof and floor rocks	34°
Elastic modulus of coal	2600 MPa
Poisson’s ratio of coal	0.22
Internal cohesive force of coal	2.1 MPa
Internal friction angle of coal	30°
Limiting gas adsorption amount of coal	22.2 m^3^/t
Adsorption equilibrium constant	0.68 MPa^−1^
Temperature Mass of combustible materials per volume of coal	293 K 1.07 t/m^3^
Initial porosity of coal	0.055
Initial permeability of coalbed	1.8 × 10^−15^ m^2^
Kinetic viscosity coefficient of gas	1.84 × 10^−5^ Pa.s
Density of gas	0.717 kg/m^3^
Atmospheric pressure at face	0.1 MPa
Area of roadway cross-section	14 m^2^
Bulk density	1.4 t/m^3^
Intensity of gas emission from initially collapsed coal	0.07 m^3^/t.min
Decay coefficient of gas from collapsed coal	0.079 min^−1^
Decay coefficient of gas from coal wall	0.000054 min^−1^

## Data Availability

Not applicable.
